# Effect of 6-week tadalafil treatment on blood-based biomarkers of neurodegeneration: A post-hoc analysis of a randomized controlled trial

**DOI:** 10.1177/13872877261421227

**Published:** 2026-03-13

**Authors:** Emanuel Fryk, Vagner Ramon Rodrigues Silva, Lena Strindberg, Magnus Gisslén, Henrik Zetterberg, Kaj Blennow, Per-Anders Jansson

**Affiliations:** 1 Department of Molecular and Clinical Medicine, Institute of Medicine, 70712 Sahlgrenska Academy, University of Gothenburg, SU Sahlgrenska, Gothenburg, Sweden; 2 Department of Infectious Diseases, Institute of Biomedicine, 70712 Sahlgrenska Academy, University of Gothenburg, Gothenburg, Sweden; 3 Department of Infectious Diseases, Sahlgrenska University Hospital, Region Västra Götaland, Gothenburg, Sweden; 4 Department of Psychiatry and Neurochemistry, Institute of Neuroscience and Physiology, 70712 Sahlgrenska Academy, University of Gothenburg, Mölndal, Sweden; 5 Clinical Neurochemistry Laboratory, Sahlgrenska University Hospital, Region Västra Götaland, Mölndal, Sweden; 6 Department of Neurodegenerative Disease, UCL Institute of Neurology, Queen Square, London, UK; 7 UK Dementia Research Institute at UCL, London, UK; 8 Hong Kong Center for Neurodegenerative Diseases, InnoHK, Hong Kong, China; 9 Wisconsin Alzheimer's Disease Research Center, University of Wisconsin School of Medicine and Public Health, University of Wisconsin-Madison, Madison, WI, USA; 10 Centre for Brain Research, Indian Institute of Science, Bangalore, India; 11 Paris Brain Institute, ICM, Pitié-Salpêtrière Hospital, Sorbonne University, Paris, France; 12 Neurodegenerative Disorder Research Center, Division of Life Sciences and Medicine, and Department of Neurology, Institute on Aging and Brain Disorders, University of Science and Technology of China and First Affiliated Hospital of USTC, Hefei, P.R. China

**Keywords:** Alzheimer's disease, biomarkers, neurodegeneration, randomized controlled trial, tadalafil, type 2 diabetes

## Abstract

Phosphodiesterase-5 (PDE-5) inhibitors may be beneficial in Alzheimer's disease (AD). We assessed the PDE-5 inhibitor tadalafil effect on plasma biomarkers of neurodegeneration in 15 individuals with type 2 diabetes post-hoc in a randomized placebo-controlled trial (ClinicalTrials.gov: NCT02601989) at Sahlgrenska University Hospital. Tadalafil reduced plasma amyloid-β 40 and 42 but not the 42/40 ratio over a 6-week treatment period. Glial fibrillary acidic protein was reduced, but not phosphorylated tau217, neurofilament light protein or growth/differentiation factor 15. Tadalafil reduced plasma levels of biomarkers for amyloid metabolism and astroglial activation in patients with diabetes. Designated clinical trials are warranted to validate these results.

## Introduction

Patients with type 2 diabetes have almost twice the incidence of dementia relative to other patient groups and prioritization of neurodegenerative outcomes in diabetes trials has been proposed.^1^ According to the National Institute on Aging-Alzheimer's Association, knowledge on how comorbidities and medications affect circulating brain biomarkers is a top research priority.^2^ This is of relevance when assessing the effects of pharmaceutical treatment in patients with diabetes given the bidirectional relationship between cognitive dysfunction and diabetes management.^3^

Well-established blood-based biomarkers allow for a sensitive evaluation of several aspects of the neurodegenerative spectra. Decreased concentrations of the 42-amino acid isoform of amyloid-β (Aβ_42_) is the defining marker in Alzheimer's disease (AD),^2^ and is often adjusted for by the most abundant Aβ isoform, Aβ_40_, through the Aβ ratio. Tauopathy in AD can be assessed by measuring phosphorylated tau217 (pTau217) in plasma.^4^ Aβ_42_ and pTau217 may also be used together for screening of AD through the pTau217/Aβ_42_ ratio.^5^ Neurofilament light protein (NfL) is a sensitive yet nonspecific marker of neurodegeneration and axonal degeneration^6^ and glial fibrillary acid protein (GFAP) is a prototypical marker of astrocytic activation, which increases over the AD continuum.^7^ Furthermore, growth/differentiation factor 15 (GDF-15) is a cellular stress marker related to metabolic disease and inflammation.^8^

A longitudinal cohort study recently reported that treatment with a phosphodiesterase-5 (PDE-5) inhibitor is associated with a lower incidence of AD.^9^ A phase III randomized controlled clinical trial (RCT) of a PDE-5 inhibitor is ongoing (ClinicalTrials.gov, NCT05531526). In a recent placebo-controlled RCT in patients with diabetes, we showed that chronic treatment with the PDE-5 inhibitor tadalafil lowered mean blood glucose as measured by hemoglobin A1c (HbA1c).^10^ In this post-hoc analysis of the RCT, we hypothesized that tadalafil would modulate blood-based biomarkers of neurodegeneration and cellular stress in apparently cognitive healthy patients with diabetes.

## Methods

### Participants

**Figure 1.**
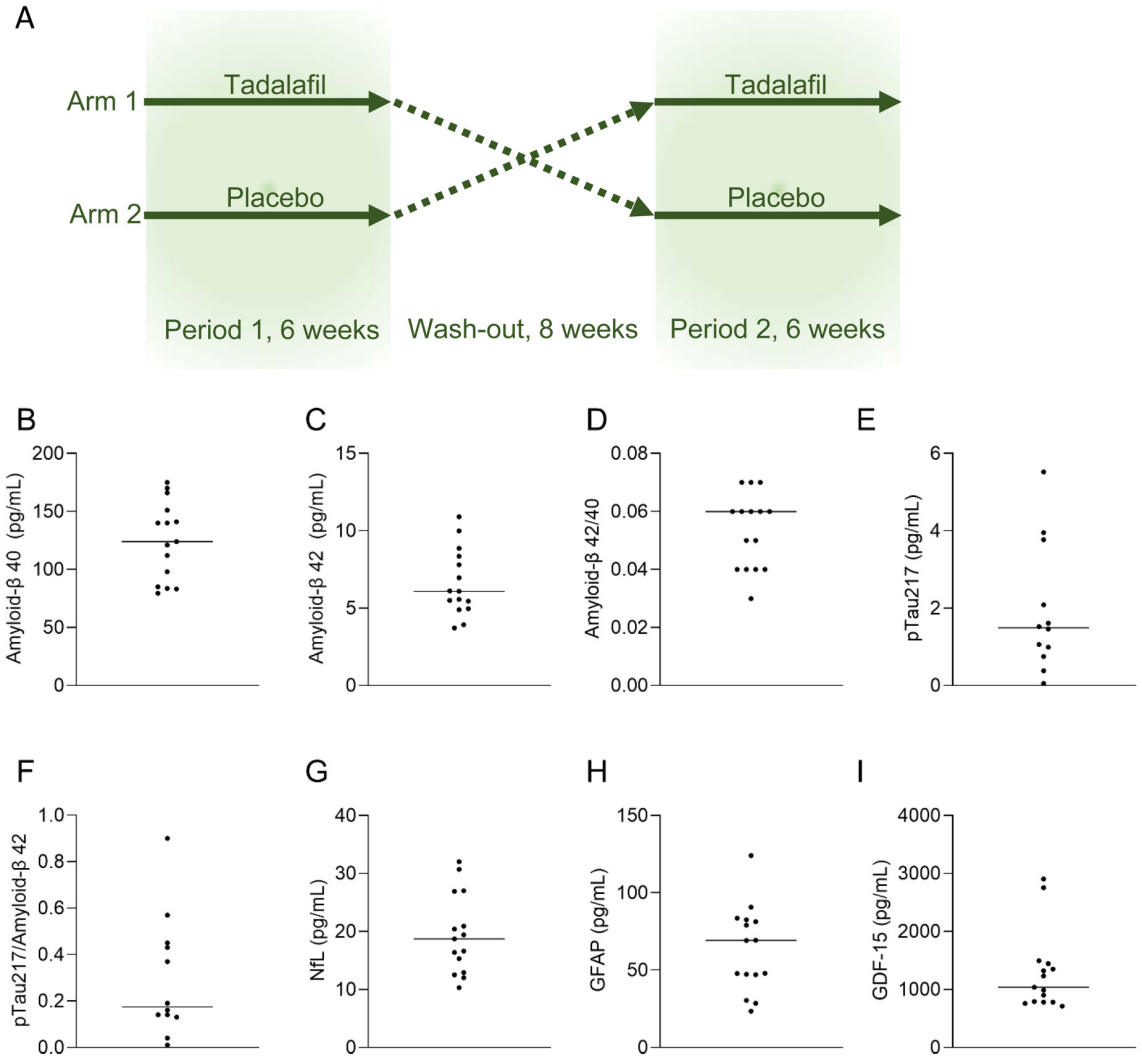
Schematic illustration of the MAKROTAD study.^10^ Patients with type 2 diabetes were randomized to start with either 6-week daily administration of 20 mg tadalafil, or placebo. After 8 weeks of washout, participants shifted treatment for the second treatment period of 6-weeks. Fasting venous blood samples were collected before and after each treatment period (A). Biomarkers of neurodegeneration at randomization for participants in the per protocol analysis (before treatment, n = 15, except pTau217, n = 12), line indicates median value (B-H). pTau217: phosphorylated Tau217; NfL: neurofilament light protein; GFAP: glial fibrillary acid protein; GDF-15: growth/differentiation factor 15.

In this post-hoc analysis, we measured biomarkers of neurodegeneration and cellular stress in plasma samples from fifteen individuals with diabetes who completed a double-blind, randomized, placebo-controlled, cross-over phase 2 trial using tadalafil 20 mg once daily over 6 weeks per-protocol (ClinicalTrials.gov, NCT02601989) (Figure 1a).^10^ Of the 23 individuals originally enrolled, 15 completed the trial with attendance at all visits, daily use of the study medication and valid measurements for all pre-registered endpoints. Participants were examined at Sahlgrenska University Hospital and the Wallenberg Laboratory, Sahlgrenska Academy, University of Gothenburg, between 22 January 2016 and 31 January 2019. Cognitive impairment symptoms were exclusion criteria as they could negatively affect compliance with the study protocol.

### Measurements

At an initial screening visit, participants were interviewed about their health and lifestyle habits. At the start (baseline) and end of each 6-week treatment period, participants attended the clinic after an overnight fast for measurements including weight, height, waist circumference, blood pressure, and venous blood sampling. Plasma was stored at −80 °C awaiting analysis. Blood HbA1c, plasma glucose, cholesterol and lipoprotein cholesterols, triglycerides, creatinine, alanine aminotransferase (ALT) and aspartate aminotransferase (AST) in serum and creatinine/albumin ratio in urine were analyzed with accredited methods at the Clinical Chemistry Laboratory at Sahlgrenska University Hospital, Gothenburg. Plasma Aβ_40_, Aβ_42_, NfL, and GFAP concentrations were measured using a commercially available Single molecule array (Simoa) assay on the HD-X platform (Quanterix, Billerica, MA), pTau217 with the Lumipulse immunoassay (Fujirebio) and GDF-15 using an Elecsys electrochemiluminescence immunoassay on the Cobas platform (Roche Diagnostics, Rotkreuz, Switzerland). A single batch of reagents was used, and all measurements were performed in a single round of experiments by board-certified laboratory technicians blinded to clinical data and treatments. Intra-assay coefficients of variation were below 10% for all analytes. For pTau217, plasma was available for 12 and 9 individuals at baseline and follow-up, respectively, and levels were below the limit of quantification (1.2 pg/mL) in 43% of the samples. Measurements below level of quantification were also included in the analysis and were set to 0.05. Biomarkers of neurodegeneration were analyzed at the Neurochemistry Laboratory, Sahlgrenska University Hospital, Mölndal, Sweden.

### Statistics

Data in Table 1 is presented as n (%) for categorical variables and mean (SD) of continuous variables for participants in the per protocol analysis. For treatment outcomes in Table 2, period-adjusted p-values were calculated by standard cross-over analysis method using Fisher's non-parametric permutation test. The limited sample size did not allow for adjustment of confounding variables. SAS 9.4 by SAS Institute Inc., Cary, NC, USA was used for the analyses.

**Table 1.** Characteristics of study participants.

Male	10 (66.7%)
Age, y	64.8 (4.6)
Body mass index, kg/m^2^	31.5 (3.9)
Waist, cm	109 (9)
Systolic blood pressure, mmHg	148 (14)
Diastolic blood pressure, mmHg	88 (8)
Diabetes duration, y	4.0 (2.9)
Former smoker	9 (60.0%)
fP-Glucose, mmol/L	6.5 (1.3)
B-HbA1c, mmol/mol	45 (5)
S-Cholesterol, mmol/L	5.1 (1.0)
S-LDL cholesterol, mmol/L	3.4 (1.0)
S-HDL cholesterol, mmol/L	1.2 (0.3)
S-Triglycerides, mmol/L	2.0 (0.7)
S-Creatinine, µmol/L	78 (18)
S-ALT, µkat/L	0.62 (0.23)
S-AST, µkat/L	0.46 (0.12)
U-Albumin, mg/L	42 (63)

Data are presented as n (%) for categorical variables and mean (SD) of continuous variables for participants in the per protocol analysis, n = 15 (10).

ALT: alanine aminotransferase; AST: aspartate aminotransferase; B: blood; fP: fasting plasma; HbA1c: hemoglobin A1c; HDL: high-density lipoprotein; LDL: low-density lipoprotein; S: serum; U: urine.

Normal reference for HbA1c ≤ 48 mmol/mol.

**Table 2.** Change in mean (95% CI) biomarker concentrations following treatment with tadalafil or placebo.

Variable	Placebo	Tadalafil 20 mg	Period adjusted mean difference, tadalafil vs. placebo	Period adjusted p-value
Body mass index, kg/m^2^	0.085 (−0.100; 0.271)	0.121 (−0.033; 0.280)	0.004 (−0.200; −0.217)	1.00
Systolic blood pressure, mmHg	−0.267 (−7.333; 6.417)	0.467 (−7.167; 8.000)	2.15 (−7.33; 11.81)	0.65
Diastolic blood pressure, mmHg	1.93 (−1.42; 5.31)	−2.90 (−5.86; −0.10)	−4.10 (−8.75; 0.50)	0.079
fP-Glucose, mmol/L	−0.247 (−0.740; 0.262)	0.086 (−0.550; 0.850)	0.570 (−1.500; 0.383)	0.27
B-HbA1c, mmol/mol	0.933 (−0.333; 2.200)	−1.93 (−2.86; −1.00)	−2.40 (−4.00; 0.83)	**0** **.** **010**
Amyloid-β 40, pg/mL	8.92 (−1.05; 18.98)	−6.21 (−18.45; 5.25)	−22.0 (−36.2; −8.05)	**0**.**0072**
Amyloid-β 42, pg/mL	0.493 (−0.100; 1.084)	−0.454 (−1.356; 0.382)	−1.30 (−2.25; −0.36)	**0**.**015**
Amyloid-β 42/40	−0.000 (−0.003; 0.002)	0.000 (−0.003; 0.003)	0.000 (−0.004; 0.005)	0.87
pTau217, pg/mL*	0.427 (−0.038; 0.885)	0.162 (−0.103; 0.418)	−0.036 (−0.515; 0.320)	0.66
pTau217/Amyloid-β 42 *	0.073 (−0.028; 0.186)	0.039 (−0.014; 0.089)	−0.008 (−0.101; 0.085)	0.93
NfL, pg/mL	2.14 (−0.62; 4.83)	0.053 (−2.460; 2.683)	−2.46 (−6.73; 1.25)	0.18
GFAP, pg/mL	5.07 (−4.20; 15.20)	−5.54 (−16.73; 5.19)	−14.6 (−27.8; −0.18)	**0**.**048**
GDF-15, pg/mL	−46.9 (−256.8; 155.9)	4.94 (−162.69; 136.91)	15.9 (−124.9; 160.06)	0.83

Period-adjusted p-value was calculated by standard cross-over analysis method using Fisher's non-parametric permutation test. B: blood; fP: fasting plasma; GDF-15: growth/differentiation factor 15; GFAP: glial fibrillary acidic protein; NfL: neurofilament light protein; pTau217: phosphorylated Tau217. Statistical significance was defined as *p* < 0.05, n = 15.

*n = 9 in longitudinal analysis.

Statistical significance in bold was defined as *p* < 0.05.

## Results

### Characteristics of study participants

At baseline, participants had the following mean values: HbA1c 45 mmol/mol, BMI 31.5 kg/m^2^, blood pressure 148/88 mmHg, and LDL cholesterol 3.4 mmol/L (Table 1). Baseline levels of Aβ_42/40_, pTau217, pTau217/Aβ_42_, NfL, GFAP, and GDF-15 are presented in Figure 1.

### Effects of tadalafil treatment

Tadalafil treatment, compared with placebo, did not alter BMI, blood pressure or fasting plasma glucose, but decreased HbA1c, as previously shown in the full analysis set.^10^ Tadalafil also decreased plasma levels of Aβ_40_ and Aβ_42_ but did not affect the Aβ_42/40_ ratio, pTau217 or the pTau217/Aβ_42_ ratio in plasma (Table 2). Further, tadalafil decreased the plasma level of GFAP, while plasma levels of NfL and GDF-15 did not change compared to placebo. Changes in Aβ_40_, Aβ_42_, Aβ_42/40_, pTau217, pTau217/Aβ_42_, NfL, and GFAP did not correlate with changes in HbA1c when comparing tadalafil and placebo. However, a moderate inverse correlation was observed between GDF-15 and HbA1c (Supplemental Table 1).

## Discussion

We performed a post-hoc analysis of a placebo-controlled RCT to assess the effect of 6-week daily treatment with tadalafil 20 mg on blood-based biomarkers of neurodegeneration in patients with diabetes. Tadalafil, compared with placebo, decreased plasma levels of Aβ_40_ and Aβ_42_ and the neuroinflammatory marker GFAP, but did not affect the AD-specific biomarker Aβ_42/40_ ratio or plasma levels of pTau217. The net change of the stress marker GDF-15 in plasma after tadalafil versus placebo was inversely correlated with the net change of HbA1c.

Our observation that plasma levels of Aβ_40_ and Aβ_42_, but not the Aβ_42/40_ ratio, were decreased by tadalafil suggests that the observed effects of tadalafil are not specific for AD plaque pathology. However, the results may indicate an effect on amyloidogenic processing of AβPP, the precursor protein of Aβ.^11^ It is also possible that improvements in microcirculation induced by tadalafil could improve clearance of Aβ_40_ and Aβ_42_, or that tadalafil modulates inflammatory effects with secondary effects on both Aβ_40_ and Aβ_42_.^12^ Previous studies on comorbidities in AD have reported that diabetes is associated with increased levels of Aβ_40_ and Aβ_42_, but not the Aβ_42/40_ ratio in plasma.^13^ It is possible that the decrease in Aβ induced by tadalafil normalizes this increase.^13^ Studies in animal models have previously suggested anti-neuroinflammatory effects of tadalafil related to amyloid pathology, and tadalafil has been shown to modulate both mTOR and NF-κB expression in the rodent brain.^14^ Similarly, a dual PDE-4/PDE-5 inhibitor has been shown to reduce activation of astrocytes and increase neuron numbers and mitigate neuroinflammation through the NF-κB and JNK pathways in cell studies and mice.^15^ Here, tadalafil also decreased levels of the astrocyte marker GFAP, indicating an improvement in neuroinflammation. This is of interest as it has previously been reported that an increased circulating GFAP level among patients with diabetes and obesity correlates with a decrease in incident mild cognitive impairment.^16^ Additional experiments should be conducted to further enhance the understanding of our observations in this clinical trial.

Baseline NfL levels were slightly higher than levels seen in healthy individuals but comparable with levels previously reported in patients with chronic kidney disease.^17^ Notably, the three individuals with the highest NfL levels also showed the highest pTau217 concentrations, indicating ongoing neuronal injury.

Furthermore, we did not observe any association between NfL and HbA1c. This may be explained by a low prevalence of complications, such as peripheral neuropathy, in our study cohort.^10^ Several studies have recognized the influence of somatic comorbidities on the interpretation of results using blood-based biomarkers of neurodegeneration.^13,17,18^ Furthermore, we have previously shown that the neprilysin inhibitor sacubitril, which is commonly used for the treatment of heart failure, skews levels of AD blood biomarkers based on Aβ, either in a ratio or by itself.^19^ This type of interference will be important as AD biomarkers enter clinical diagnostic use because it could lead to misdiagnosis. In our study, neither treatment with tadalafil nor modification of glycemia in patients with diabetes altered the Aβ_42/40_ ratio, pTau217, or NfL biomarker levels; thus, these conditions do not confound clinical interpretation of these AD biomarkers. While not seen in this study it is possible that other ratios that include Aβ_42_, e.g., the plasma pTau217/Aβ_42_ ratio, could be affected in some circumstances.^5^ A clinical consequence of our finding of decreased plasma Aβ_42_ levels is therefore that tadalafil may be another drug affecting AD blood biomarkers that include plasma Aβ.^20^

Suppression of HbA1c during tadalafil treatment was associated with increased levels of the cellular stress marker GDF-15 levels in plasma. This observation is in agreement with proposed mechanisms behind the beneficial metabolic effects of GDF-15.^8^

We report several novel observations in this post-hoc analysis from a small RCT. A limitation is that the study was not specifically powered for the outcomes of this sub-study and the small sample size did not allow for adjustment of confounding variables. However, we did not observe any significant changes in BMI and blood pressure over the treatment period. The clinical significance of our findings requires future validation, but they may be helpful to guide future research. Furthermore, to improve current treatment strategies in diabetes and neurodegenerative conditions, it is important that data from clinical trials are shared regardless of the study outcome.^21^ Unfortunately, we did not assess cognitive performance pre- and postintervention, and therefore it was not possible to evaluate whether differences in cognition were associated with changes in biomarkers. Our participants were cognitively healthy, and it is possible that tadalafil could have other or more pronounced effects in individuals with cognitive disease. Here, our pTau217 measurements were very low, often below limit of quantification. There was no indication that tadalafil would increase pTau217, but any lowering effect would be missed due to the low measurements.

In conclusion, daily tadalafil treatment in cognitively healthy individuals with diabetes lowered specific blood-based biomarkers of neurodegeneration. Designated randomized clinical trials are warranted to validate the effect of PDE-5 inhibitors on circulating biomarkers of neurodegenerative disease in diabetes patients.

## Supplemental Material

sj-docx-1-alz-10.1177_13872877261421227 - Supplemental material for Effect of 6-week tadalafil treatment on blood-based biomarkers of neurodegeneration: A post-hoc analysis of a randomized controlled trial Supplemental material, sj-docx-1-alz-10.1177_13872877261421227 for Effect of 6-week tadalafil treatment on blood-based biomarkers of neurodegeneration: A post-hoc analysis of a randomized controlled trial by Emanuel Fryk, Vagner Ramon Rodrigues Silva, Lena Strindberg, Magnus Gisslén, Henrik Zetterberg, Kaj Blennow and Per-Anders Jansson in Journal of Alzheimer's Disease

## References

[1] van Sloten TT Luchsinger JA Launer LJ , et al. Call for effective therapies for preventing dementia in people with type 2 diabetes. Lancet Diabetes Endocrinol 2024; 12: 510–513. 38901446 10.1016/S2213-8587(24)00158-X

[2] Sperling RA Aisen PS Beckett LA , et al. Toward defining the preclinical stages of Alzheimer's disease: recommendations from the national institute on aging-Alzheimer's association workgroups on diagnostic guidelines for Alzheimer's disease. Alzheimers Dement 2011; 7: 280–292. 21514248 10.1016/j.jalz.2011.03.003 PMC3220946

[3] O'Bryant SE Petersen M Hall J , et al. Medical comorbidities and ethnicity impact plasma Alzheimer's disease biomarkers: important considerations for clinical trials and practice. Alzheimers Dement 2023; 19: 36–43. 35235702 10.1002/alz.12647 PMC13270989

[4] Palmqvist S Warmenhoven N Anastasi F , et al. Plasma phospho-tau217 for Alzheimer's disease diagnosis in primary and secondary care using a fully automated platform. Nat Med 2025; 31: 2036–2043. 40205199 10.1038/s41591-025-03622-w PMC12176611

[5] Wang J Huang S Lan G , et al. Diagnostic accuracy of plasma p-tau217/Abeta42 for Alzheimer's disease in clinical and community cohorts. Alzheimers Dement 2025; 21: e70038. 10.1002/alz.70038 PMC11953589 40156286

[6] Ashton NJ Janelidze S Al Khleifat A , et al. A multicentre validation study of the diagnostic value of plasma neurofilament light. Nat Commun 2021; 12: 3400. 34099648 10.1038/s41467-021-23620-z PMC8185001

[7] Benedet AL Mila-Aloma M Vrillon A , et al. Differences between plasma and cerebrospinal fluid glial fibrillary acidic protein levels across the Alzheimer disease continuum. JAMA Neurol 2021; 78: 1471–1483. 34661615 10.1001/jamaneurol.2021.3671 PMC8524356

[8] Wang D Townsend LK DesOrmeaux GJ , et al. GDF15 Promotes weight loss by enhancing energy expenditure in muscle. Nature 2023; 619: 143–150. 37380764 10.1038/s41586-023-06249-4 PMC10322716

[9] Adesuyan M Jani YH Alsugeir D , et al. Phosphodiesterase type 5 inhibitors in men with erectile dysfunction and the risk of Alzheimer disease: a cohort study. Neurology 2024; 102: e209131. 10.1212/WNL.0000000000209131 PMC10890837 38324745

[10] Fryk E Rodrigues Silva VR Bauza-Thorbrugge M , et al. Feasibility of high-dose tadalafil and effects on insulin resistance in well-controlled patients with type 2 diabetes (MAKROTAD): a single-centre, double-blind, randomised, placebo-controlled, cross-over phase 2 trial. EClinicalMedicine 2023; 59: 101985. 37256099 10.1016/j.eclinm.2023.101985 PMC10225663

[11] Goate A Chartier-Harlin MC Mullan M , et al. Segregation of a missense mutation in the amyloid precursor protein gene with familial Alzheimer's disease. Nature 1991; 349: 704–706. 1671712 10.1038/349704a0

[12] Foley KE Winder Z Sudduth TL , et al. Alzheimer's disease and inflammatory biomarkers positively correlate in plasma in the UK-ADRC cohort. Alzheimers Dement 2024; 20: 1374–1386. 38011580 10.1002/alz.13485 PMC10917006

[13] Syrjanen JA Campbell MR Algeciras-Schimnich A , et al. Associations of amyloid and neurodegeneration plasma biomarkers with comorbidities. Alzheimers Dement 2022; 18: 1128–1140. 34569696 10.1002/alz.12466 PMC8957642

[14] Franca MER Ramos R Oliveira WH , et al. Tadalafil restores long-term memory and synaptic plasticity in mice with hepatic encephalopathy. Toxicol Appl Pharmacol 2019; 379: 114673. 31323263 10.1016/j.taap.2019.114673

[15] Zhao L Jiang W Cao Y , et al. A novel phosphodiesterase 4/5 dual-target inhibitor ameliorates neuroinflammation via the NF-kappaB and JNK pathway. Int J Biol Macromol 2025; 318: 145007. 40482756 10.1016/j.ijbiomac.2025.145007

[16] Mielke MM Evans JK Neiberg RH , et al. Alzheimer disease blood biomarkers and cognition among individuals with diabetes and overweight or obesity. JAMA Netw Open 2025; 8: e2458149. 10.1001/jamanetworkopen.2024.58149 PMC11803481 39913137

[17] Dittrich A Ashton NJ Zetterberg H , et al. Association of chronic kidney disease with plasma NfL and other biomarkers of neurodegeneration: the H70 birth cohort study in Gothenburg. Neurology 2023; 101: e277–e288. 10.1212/WNL.0000000000207419 PMC10382262 37225431

[18] Hansson O Edelmayer RM Boxer AL , et al. The Alzheimer's association appropriate use recommendations for blood biomarkers in Alzheimer's disease. Alzheimers Dement 2022; 18: 2669–2686. 35908251 10.1002/alz.12756 PMC10087669

[19] Brum WS Docherty KF Ashton NJ , et al. Effect of neprilysin inhibition on Alzheimer disease plasma biomarkers: a secondary analysis of a randomized clinical trial. JAMA Neurol 2024; 81: 197–200. 38109077 10.1001/jamaneurol.2023.4719 PMC10728797

[20] Tanne JH . FDA Approves blood test to diagnose Alzheimer's. Br Med J 2025; 389: r1082. 10.1136/bmj.r1082 40409786

[21] Knopman DK Gross RA . A call for “negative” outcome studies. Neurology 2014; 82: 11–12. 24379096 10.1212/01.wnl.0000438233.78964.77

