# Supplementary material for: Effect of 6-week tadalafil treatment on blood-based biomarkers of neurodegeneration: A post-hoc analysis of a randomized controlled trial
Source: J Alzheimers Dis. 2026 Mar 13;110(3):1072–8. doi: 10.1177/13872877261421227 (PMC13022012; doi:10.1177/13872877261421227)
Supplement: sj-docx-1-alz-10.1177_13872877261421227 - Supplemental material for Effect of 6-week tadalafil treatment on blood-based biomarkers of neurodegeneration: A post-hoc analysis of a randomized controlled trial [file sj-docx-1-alz-10.1177_13872877261421227.docx]

**Supplemental Material**

**Effect of 6-week tadalafil treatment on blood-based biomarkers of neurodegeneration: A post-hoc analysis of a randomized controlled trial**

| **Supplementary Table 1.** Spearman correlations between net change of HbA1c and net change of biomarkers during treatment with tadalafil or placebo in patients with type 2 diabetes (n=15). | | |
| --- | --- | --- |
|  | R | *p* |
| Amyloid-β 40, pg/mL | -0.43 | 0.109 |
| Amyloid-β 42, pg/mL | -0.27 | 0.323 |
| Amyloid-β 42/40 | 0.20 | 0.467 |
| pTau217, pg/mL* | 0.50 | 0.172 |
| NfL, pg/mL | -0.28 | 0.318 |
| GFAP, pg/mL | 0.16 | 0.559 |
| GDF-15, pg/mL | -0.56 | **0.029** |
| Statistical significance was defined as *p*<0.05, n=15.  GDF-15: growth/differentiation factor 15; GFAP: glial fibrillary acidic protein; NfL: neurofilament light protein | | |
| *n=9 | | |
